# Predicting the Development of Gastric Neoplasms in a Healthcare Cohort by Combining *Helicobacter pylori* Antibodies and Serum Pepsinogen: A 5-Year Longitudinal Study

**DOI:** 10.1155/2018/8796165

**Published:** 2018-07-24

**Authors:** Min-Sun Kwak, Goh Eun Chung, Su Jin Chung, Seung Joo Kang, Jong In Yang, Joo Sung Kim

**Affiliations:** Department of Internal Medicine, Healthcare Research Institute, Healthcare System Gangnam Center, Seoul National University Hospital, Seoul, Republic of Korea

## Abstract

**Background:**

*Helicobacter pylori (HP)* and gastric atrophy are risk factors for gastric cancer. We evaluated whether the combination of serum HP antibody and pepsinogen (PG), which is indicative of gastric atrophy, could serve as a predictive marker for the development of gastric neoplasms in a Korean population.

**Methods:**

The subjects who had undergone health-screening examination with endoscopic follow-ups were classified into the following 4 groups according to serum PG status and *HP* antibody at baseline: group A (*HP* (−), normal PG), group B (*HP* (+), normal PG), group C (*HP* (+), atrophic PG), and group D (*HP* (−), atrophic PG). We compared the development of gastric neoplasms among the groups.

**Results:**

Of the 3297 subjects, 1239 (37.6%) were categorized as group A, 1484 (45.0%) as group B, 536 (16.3%) as group C, and 38 (1.2%) as group D. During the 5.6 years of mean follow-up period, the annual incidence of gastric neoplasms increased gradually by 0.06% in group A, 0.16% in group B, 0.38% in group C, and 0.49% in group D. A Cox proportional hazard model showed increased development of gastric neoplasms according to group (*P* for trend = 0.025). Compared to group A, the hazard ratio was 8.25 for group D (95% confidence interval 0.2–74.24), 5.35 for group C (1.68–17.05), and 2.65 for group B (0.86–8.14).

**Conclusion:**

The combination of serum PG and *HP* antibody is useful for predicting the development of gastric neoplasms, including cancer and adenoma, in a Korean population using endoscopic surveillance.

## 1. Introduction

Gastric cancer is one of the major causes of cancer-related death worldwide, and approximately 990,000 cases of gastric cancer are diagnosed annually [1]. In Eastern Asia, including Japan and South Korea, gastric cancer is the most prevalent cancer [2]. According to Correa's cascade, multiple processes, which are known as the gastritis–atrophy–metaplasia–dysplasia–cancer sequence, are responsible for the development of the intestinal type of gastric cancer, which is thought to represent a major route of stomach carcinogenesis in Eastern Asia [3, 4].

*Helicobacter pylori (HP)* infections and the associated chronic atrophic gastritis (CAG) are two well-known major risk factors for the development of gastric cancer [5, 6]. Previous studies have typically assessed gastric atrophy by measuring the pepsinogen (PG) levels in serum samples [7, 8]. Both PG I and II are produced by chief cells and mucous neck cells of the stomach, but PG II is also produced by pyloric gland cells [9, 10]. As gastric atrophy develops, chief cells are replaced by pyloric glands, leading to a decrease in the levels of PG I, while the levels of PG II remain relatively unaffected. Therefore, both low serum PG I and a low PG I/II ratio are recognized as serological markers of gastric atrophy [11].

In Japan, the ABCD prediction model, which combines the *HP* serology test and serum PG test, has been widely used to stratify the general population according to the risk of stomach cancer. This method is simple and less invasive than esophagogastroduodenoscopy. In the ABCD method, individuals are classified into four groups as follows: (1) immunoglobulin G (IgG) anti-*HP* antibody-negative and normal PG level (group A), (2) IgG anti-*HP* antibody-positive and normal PG level (group B), (3) IgG anti-*HP* antibody-positive and atrophic PG test (group C), and (4) IgG anti-*HP* antibody-negative and atrophic PG test (group D). A previous cross-sectional study revealed an increasing trend of gastric cancer in the order of group A to group D [8]. In Japan, a prospective study also demonstrated that the ABCD method predicts the development of gastric cancer [7]. In a recent meta-analysis, this four-risk group model, which combines the serum PG test and *HP* antibodies, was shown to categorize risk-stratified asymptomatic adults into the four risk groups of incident gastric cancer with moderate accuracy [6].

However, the ABCD method has several limitations. First, most studies were performed only in Japan [6], and there is a racial-ethnic difference in the occurrence of gastric cancer [12]. Second, the ABCD method was shown to be associated with gastric neoplasms, including not only gastric cancer but also gastric adenoma in cross-sectional analysis [13]. Because most premalignant gastric lesions can be treated with endoscopic treatment, evaluating the applicability of the ABCD method to predict not only gastric cancer but also gastric adenoma in a longitudinal study is important. Thus, this longitudinal cohort study aimed to evaluate whether or not the ABCD method, which combines serum PG and *HP* antibody tests, could predict the development of gastric cancer and gastric adenoma in a healthy Korean population using an annual or biennial endoscopic follow-up.

## 2. Methods

### 2.1. Study Population

**Figure 1:**
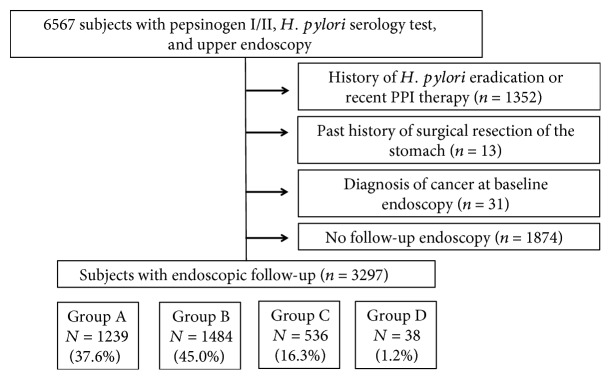
Flowchart of subjects in this study.

In total, 6567 subjects who had undergone serum PG and *HP* IgG antibody testing and esophagogastroduodenoscopy on the same day during a health-screening examination at Seoul National University Hospital Gangnam Center between March 2008 and December 2009 were initially included. Overall, 1352 subjects with a prior history of *HP* eradication or recent proton-pump inhibitor therapy 1 month prior to enrollment and 13 subjects with a past history of gastric surgery were excluded. Thirty-one subjects who were diagnosed with gastric cancer at baseline and 1874 subjects without any follow-up endoscopy were also excluded (Figure 1). Subjects were encouraged to undergo an endoscopic examination annually to screen for the development of stomach cancer, and these follow-up data were analyzed in this study.

This study protocol conformed to the ethical guidelines of the 1975 Declaration of Helsinki and was approved by the Institutional Review Board of Seoul National University Hospital (1504-044-663). The need for informed consent was waived by the Institutional Review Board of Seoul National University Hospital because the researchers accessed only deidentified databases for analytical purposes.

### 2.2. Serum *HP* IgG Antibody Assay

Anti-*HP* antibody IgG (anti-*HP* Ab IgG) was measured using an enzyme-linked immunosorbent assay kit (Radim Diagnostics, Rome, Italy) and an automatic analyzer, Alisei® (Seac, Pomezia, Italy), which was previously validated in a nationwide Korean seroepidemiologic study [14, 15]. Anti-*HP* levels higher than 15 RU/mL were considered positive.

### 2.3. Serum PG Levels

Serum levels of PG I and II were measured using a latex-enhanced turbidimetric immunoassay (HBi Corp., Seoul, Korea, imported from Shima Laboratories, Tokyo, Japan), and the PG I-to-PG II ratios (PG I/II) were calculated. Serum PG status was defined as “atrophic” when both criteria of a serum PG I level ≤ 70 ng/mL and a PG I/II ratio ≤ 3.0 were simultaneously fulfilled, which is the most widely used definition [16]. All other cases were classified as “normal.”

### 2.4. Classification of Subjects according to the ABCD Method

Subjects were classified into 4 groups according to the prediction by the ABCD method, which combined the serum PG status and *HP* antibody testing. According to the original ABC method, “atrophy” was defined as PGI ≤ 70 ng/mL and PG I/II ≤ 3 [7, 17]. Subjects were divided as follows: group A (*HP* (−), normal PG status), group B (*HP* (+), normal PG status), group C (*HP* (+), atrophic PG status), and group D (*HP* (−), atrophic PG status).

### 2.5. Endoscopic Examination and Follow-Up

Fifteen experienced board-certified endoscopists performed esophagogastroduodenoscopy using GIF-H260 (Olympus, Tokyo, Japan), EG-405WR5, or EG-590WR (Fuji-non, Saitama, Japan). A follow-up endoscopy was recommended within 1 or 2 years. The endoscopists performed the endoscopic examination without knowledge of the serological data of the subjects. A gastric biopsy was performed when a lesion was suspected to be gastric cancer, and the biopsies were examined by expert gastrointestinal pathologists according to the World Health Organization (WHO) criteria [18]. Gastric adenoma was considered low-grade adenoma or high-grade adenoma according to the Vienna classification [19]. Gastric cancer was classified as a differentiated type (including well or moderately differentiated adenocarcinomas) and undifferentiated type (including poorly differentiated, signet-ring cell, and mucinous carcinomas) [20]. Gastric cancer was also classified according to Lauren's criteria as intestinal and diffuse types [21].

### 2.6. Statistical Analysis

The primary outcome in this study was the development of gastric cancer or adenoma. The data are expressed as the mean ± standard deviation or median (interquartile range) for continuous variables and frequency (%) for categorical variables. Analysis of variance (ANOVA) was used to analyze the continuous variables, and a Kruskal-Wallis test with Bonferroni's correction was used to analyze the categorical variables. The Kaplan-Meier method and Cox proportional hazard regression analysis were used to evaluate the development of gastric cancer or high-grade adenoma. All statistical analyses were conducted using SPSS 19 (SPSS Inc., Chicago, IL, USA). A two-tailed *P* value < 0.05 was considered statistically significant.

## 3. Results

### 3.1. Baseline Characteristics

In total, 3297 subjects were included in the analysis.  Table 1 shows the baseline characteristics of the study subjects. The mean age was 51.3 years, and 70.5% of the subjects were male. Of the 3297 subjects, 1239 (37.6%) were categorized as group A, 1484 (45.0%) were categorized as group B, 536 (16.3%) were categorized as group C, and 38 (1.2%) were categorized as group D. The mean follow-up duration was 5.6 years.

### 3.2. Development of Gastric Neoplasms according to the HP Antibody and Serum PG Levels

Table 2 shows the development of gastric neoplasms during the follow-up period according to the groups. A total of 15 gastric cancers and 14 gastric adenomas developed among the 3297 subjects during the follow-up period. The mean age at diagnosis was 56.8 years, and 23 subjects (79.3%) were male. The annual incidence rate of gastric neoplasms was 0.16%/year. Most gastric cancers were the intestinal type (12/15, 80%), and diffuse-type cancers developed in only 3 subjects (3/15, 20%). Regarding adenoma, 71.4% of the total cases developed low-grade adenoma. The annual incidence rate of gastric cancer or gastric adenoma also increased according to the ABCD group classification by 0.06% in group A, 0.16% in group B, 0.38% in group C, and 0.49% in group D.

The details regarding the incidental gastric neoplasms in the *HP*-negative subjects (group A) in this study are presented in Supplementary Table 1. One case exhibited a new *HP* infection during the follow-up period (1 year after enrollment) and developed high-grade adenoma 6 years later. The other female subjects had developed signet-ring cell carcinoma. Two cases with low-grade adenoma showed no evidence of pathological and serological *HP* infections during the follow-up period, and both cases were successfully treated with endoscopic mucosal resection.

### 3.3. Prediction of the Development of Gastric Neoplasms according to the ABCD Group

**Figure 2:**
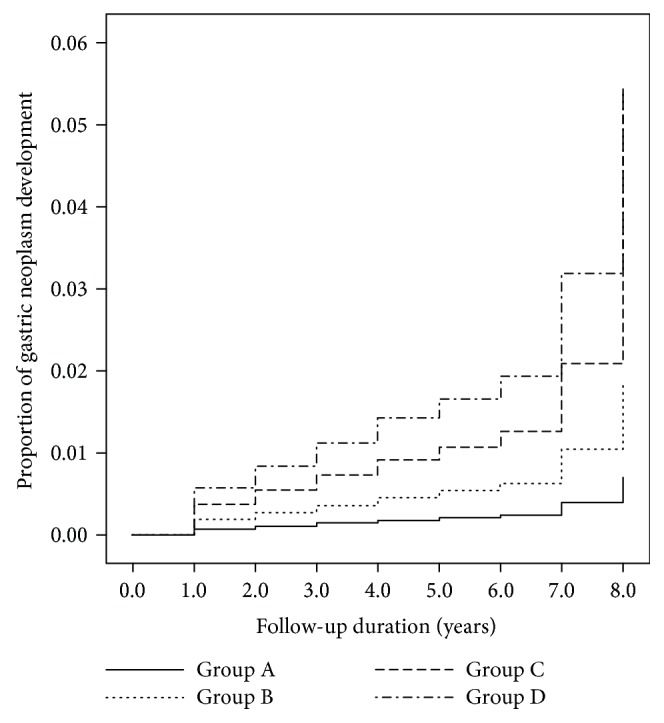
Incidence of gastric neoplasm according to the groups. This figure shows Cox regression analysis for the incidence of gastric cancer and gastric adenoma according to the groups (classified by *H. pylori* antibody status and pepsinogen status).

The Cox proportional hazard model (Table 3, Figure 2) showed an increased development of gastric neoplasms, including gastric cancer and adenoma, according to the group (*P* for trend = 0.025). Compared to group A, the hazard ratio was 8.25 for group D (95% confidence interval (CI) 0.2–74.24), 5.35 for group C (95% CI 1.68–17.05), and 2.65 for group B (0.86–8.14).

**Figure 3:**
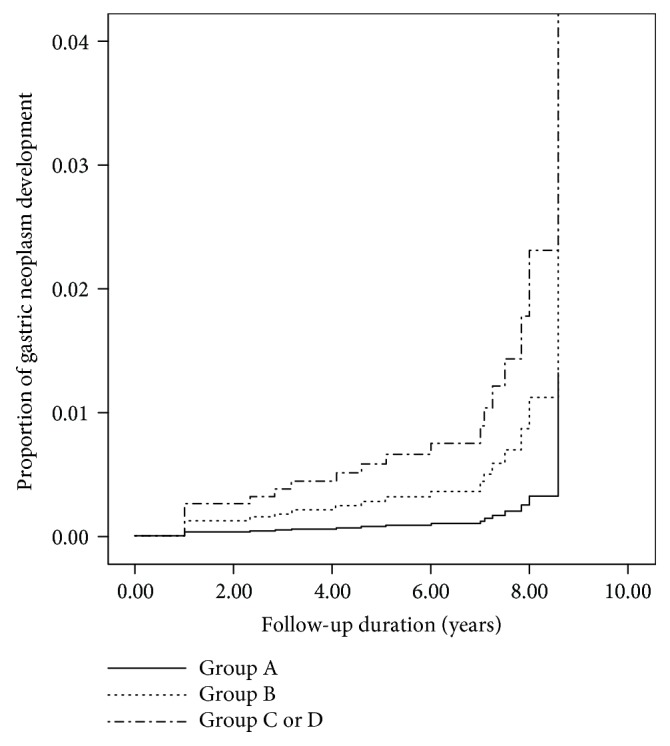
Incidence of gastric cancer and high-grade adenoma according to the groups. This figure shows Cox regression analysis for the incidence of gastric cancer and high-grade gastric adenoma according to the groups (classified by *H. pylori* antibody status and pepsinogen status).

By considering only gastric cancer or high-grade adenoma, the Cox proportional hazard model (Table 4, Figure 3) showed an increased development of gastric cancer or high-grade adenoma according to the group (*P* for trend = 0.040). Compared to group A, the hazard ratio was 7.10 for groups C or D (95% confidence interval (CI) 1.48–34.01, *P* = 0.042) and 3.45 for group B (95% CI 0.74–16.01).

## 4. Discussion

This study showed an increased development of gastric neoplasms (including gastric adenoma and gastric cancer) in the order of group A (*HP* Ab (−)/atrophy (−) group) to group B (*HP* Ab (+)/atrophy (−) group), group C (*HP* Ab (+)/atrophy (+) group), and group D (*HP* Ab (–)/atrophy (+) group) during the follow-up period (mean of 5.6 years). To date, most studies evaluating the usefulness of the ABCD method have been performed in Japan, and this study confirmed the usefulness of the ABCD method in Korea, which is another area with a high prevalence of gastric cancer. Therefore, the ABCD method, which is noninvasive and conveniently combines the *HP* IgG antibody and serum PG levels, is useful for risk stratification of the development of gastric neoplasms in a Korean population.

Consistent with previous studies, compared to subjects in group A, the highest gastric neoplasm incidence was observed in group D, followed by group C and then group B with hazard ratios of 8.25, 5.35, and 2.65, respectively. Although there was a higher tendency of cancer development in group B than in group A, there was no statistically significant difference between these two groups (*P* = 0.088). Thus, the development of gastric cancer requires time in subjects with *HP* infections without atrophy because *HP*-induced gastric cancer usually develops through a gastritis-atrophy-metaplastic change. Previous studies with longer follow-up periods (more than 10 years) showed a significant difference in gastric cancer development between groups A and B [22], but studies with shorter follow-up periods showed a higher but not significant difference between group A and group B [7, 23].

In contrast to previous studies, the participants in group D did not develop gastric cancer or high-grade adenoma. In the present study, only 1 low-grade adenoma occurred in group D. However, this finding should not be considered evidence that the risk in group D is low in the Korean population because only a small number of subjects were categorized as group D (*n* = 38, 1.2%). In previous studies, the proportion of group D ranged from 0.7 to 6.3% [7, 22–24], and the proportion of group D was relatively low in this study. This finding might be related to a lower seroclearance of *HP* Ab in Korean subjects or an earlier development of stomach cancer in Korean subjects before the seroclearance of *HP* Ab. A previous meta-analysis similarly failed to show a significant difference between group C and group D [6]. A recent 20-year prospective study in Hisayama showed that the cumulative incidence of gastric cancer was significantly increased in groups B, C, and D. However, no significant difference was found between groups C and D, which is consistent with our results [22]. Thus, more large-scale long-term studies should be performed to draw a confirmative conclusion.

Although the incidence rate was extremely low, gastric cancer developed in group A. *HP*-negative gastric cancer is rare, is most likely to be a diffuse type, and lacks male dominancy [25]. Group A has been recently shown to include not only “true group A” but also subjects with endoscopic atrophy with a previous history of *HP* eradication and a spontaneous resolution of a previous *HP* infection [26–28]. More data should be collected to define *HP*-negative stomach cancer and identify the mechanism of cancer occurrence in these subjects.

This study has several strengths. First, this study was the first Korean large-scale longitudinal study with a follow-up period longer than 5 years. Most previous studies were performed in Japan. Because the occurrence of stomach cancer shows ethnic differences, validation in other populations might be helpful for the identification of the mechanism of gastric carcinogenesis. Second, the incidence of both gastric cancer and gastric adenoma was evaluated. Third, regular endoscopic surveillance was performed in this study, while most previous prospective studies evaluated the incidence of gastric cancer based on the double-contrast barium X-ray and PG test, followed by endoscopy. In this case, the incidence of cancer in groups C or D could be overestimated. Fourth, a previous history of *HP* eradication or PPI therapy could be thoroughly examined using a specific questionnaire.

This study also has several limitations. First, studies with longer follow-up duration should be conducted [29, 30]. Although the follow-up duration was more than 5 years in this study, a follow-up of these patients for more than 10–15 years may increase our understanding of the predictability of these serum markers. Second, although we excluded subjects who had a history of *HP* eradication at baseline, subjects who had eradication of *HP* during the follow-up period were not excluded. Third, subjects with new *HP* infections during the follow-up period were not excluded. Fourth, we showed baseline pepsinogen levels in this study, not the change of pepsinogen level during follow-up. Further study with follow-up pepsinogen levels might give more information about the change of functional status of the stomach according to the different risk subgroups. Fifth, we could not confirm whether the serum pepsinogen test reflects the severity of histological atrophy, because we did not perform routine biopsy for evaluation of gastric mucosa damage except for the presence of endoscopic abnormal lesions. However, correlation between serological evaluation by pepsinogen test and the severity of histological damage by operative link on gastritis assessment and operative link on gastritis/intestinal-metaplasia assessment staging system is well established [31].

In conclusion, this longitudinal cohort study showed that the ABCD method, which combines serum PG and *HP* antibody tests, is useful for predicting the development of gastric neoplasms, including cancer and adenoma, in a healthy Korean population using endoscopic surveillance.

## Figures and Tables

**Table 1:** Baseline characteristics of subjects according to the group.

	Total	Group A	Group B	Group C	Group D	*P* value
Number of subjects (%)	3297 (100%)	1239 (37.6%)	1484 (45.0%)	536 (16.3%)	38 (1.2%)	
*H. pylori* Ab		Negative	Positive	Positive	Negative	
Pepsinogen		Normal	Normal	Atrophic	Atrophic	
Age (years)^a^	51.3 ± 9.4	50.0 ± 9.6	51.0 ± 8.8	55.1 ± 9.4	53.3 ± 8.3	0.039
Male sex (%)	2326 (70.5%)	854 (68.9%)	1096 (73.9%)	353 (65.9%)	22 (57.9%)	<0.001
Pepsinogen I (ng/mL)^a^	57.8 ± 29.9	49.9 ± 25.5	71.0 ± 31.7	42.5 ± 16.3	19.3 ± 12.2	<0.001
Pepsinogen II (ng/mL)^a^	14.7 ± 9.1	8.9 ± 4.8	18.0 ± 10.0	19.3 ± 7.3	11.0 ± 4.9	0.013
Pepsinogen I/II ratio^a^	4.5 ± 1.8	5.8 ± 1.4	4.3 ± 1.3	2.2 ± 0.6	1.8 ± 0.9	<0.001
Follow-up duration (years)^a^	5.6 ± 2.0	5.6 ± 1.9	5.5 ± 2.0	5.5 ± 2.0	5.4 ± 2.0	0.404
Follow-up duration (months (median, range))	80 (12–104)	81 (12–103)	79 (12–104)	79 (12–104)	77 (22–102)	0.325

^a^Mean ± SD.

**Table 2:** Characteristics of incidental gastric cancer and adenoma during follow-up according to the group.

	Total	Group A	Group B	Group C	Group D
		(*n* = 1239)	(*n* = 1484)	(*n* = 536)	(*n* = 38)
Incidence of gastric cancer	**15**	**1**	**7**	**7**	**0**
Intestinal type	12	0	6	6	0
Diffuse type	3	1	1	1	0
Incidence of gastric adenoma	**14**	**3**	**6**	**4**	**1**
Low-grade adenoma	10	2	4	3	1
High-grade adenoma	4	1	2	1	0
Annual incidence rate (%/year)	**0.16**	**0.06**	**0.16**	**0.38**	**0.49**

**Table 3:** Hazard ratio for the incidence of gastric adenoma and cancer by Cox regression analysis.

	Hazard ratio	95% confidence interval	*P* value
Group			
A	1		0.025^a^
B	2.65	0.86–8.14	0.088
C	5.35	1.68–17.05	0.005
D	8.25	0.92–74.24	0.060
Age	1.049	1.008–1.091	0.018
Male sex	1.716	0.692–4.254	0.244

^a^ *P* for trend.

**Table 4:** Hazard ratio for the incidence of high-grade gastric adenoma and cancer by Cox regression analysis.

	Hazard ratio	95% confidence interval	*P* value
Group			
A	1		0.040^a^
B	3.45	0.74–16.01	0.114
C or D	7.10	1.48–34.01	0.014
Age	1.05	1.00-1.10	0.042
Male sex	2.45	0.71–8.53	0.158

^a^ *P* for trend.

## Data Availability

The data used to support the findings of this study are available from the corresponding author upon request.

## Supplementary Materials

Supplementary Materials Table 1: characteristics of gastric cancer and adenoma in group A.
