# Supplementary material for: Predicting the Development of Gastric Neoplasms in a Healthcare Cohort by Combining Helicobacter pylori Antibodies and Serum Pepsinogen: A 5-Year Longitudinal Study
Source: Gastroenterol Res Pract. 2018 Jul 24;2018:8796165. doi: 10.1155/2018/8796165 (PMC6081561; doi:10.1155/2018/8796165)
Supplement: Supplementary Materials — Table 1: characteristics of gastric cancer and adenoma in group A. [file 8796165.f1.docx]

**Supplementary Files**

**Supplementary Table 1. Characteristics of gastric cancer and adenoma in group A**

| Age/sex | Pathology | *HP*-Ab titer ^a^ | PG I ^a^ | PG II ^a^ | PG I/II ^a^ | FU months | Atrophy ^b^ | HP eradication during FU/ FHx | FU *HP*-Ab | Pathologic *HP* |
| --- | --- | --- | --- | --- | --- | --- | --- | --- | --- | --- |
| 45/Male | High grade adenoma | 14.62 | 87.4 | 22.5 | 3.9 | 84 | O1 | -/- | Positive | Positive |
| 65/Female | Signet ring carcinoma | 7.05 | 46.9 | 7.0 | 6.7 | 34 | C3 | NA/- | Negative | NA |
| 58/Female | Tubullovillous low grade adenoma | 6.37 | 42.6 | 5.4 | 7.9 | 56 | C2 | -/- | Negative | Negative |
| 51/Male | Tubular adenoma, low grade | 2.43 | 208.6 | 30.5 | 6.8 | 84 | C2 | -/- | Negative | Negative |

PG, pepsinogen; FU, follow-up; NA, not available; FHx, family history of gastric cancer

^a^ initial value at study enrollment

^b^ Grade of atrophy on endoscopy
